# Three-dimensional Reconstruction of Peripheral Nerve Internal Fascicular Groups

**DOI:** 10.1038/srep17168

**Published:** 2015-11-24

**Authors:** Yingchun Zhong, Liping Wang, Jianghui Dong, Yi Zhang, Peng Luo, Jian Qi, Xiaolin Liu, Cory J. Xian

**Affiliations:** 1 School of Automation, Guangdong University of Technology, Guangzhou 510006, China; 2 Sansom Institute for Health Research and School of Pharmacy and Medical Sciences, University of South Australia, Adelaide, SA 5001, Australia; 3 School of Natural and Built Environments, University of South Australia, Adelaide, SA 5095, Australia; 4 Department of Plastic and Reconstructive Surgery, The First Affiliated Hospital of Sun Yat-sen University, Guangzhou, Guangdong, 510080, China; 5 Department of Bone and Joint Surgery, Shenzhen Sixth People’s Hospital, Shenzhen, Guangdong, 518000, China; 6 Department of Orthopedics trauma and Microsurgery, The First Affiliated Hospital of Sun Yat-sen University, Guangzhou, Guangdong, 510080, China

## Abstract

Peripheral nerves are important pathways for receiving afferent sensory impulses and sending out efferent motor instructions, as carried out by sensory nerve fibers and motor nerve fibers. It has remained a great challenge to functionally reconnect nerve internal fiber bundles (or fascicles) in nerve repair. One possible solution may be to establish a 3D nerve fascicle visualization system. This study described the key technology of 3D peripheral nerve fascicle reconstruction. Firstly, fixed nerve segments were embedded with position lines, cryostat-sectioned continuously, stained and imaged histologically. Position line cross-sections were identified using a trained support vector machine method, and the coordinates of their central pixels were obtained. Then, nerve section images were registered using the bilinear method, and edges of fascicles were extracted using an improved gradient vector flow snake method. Subsequently, fascicle types were identified automatically using the multi-directional gradient and second-order gradient method. Finally, a 3D virtual model of internal fascicles was obtained after section images were processed. This technique was successfully applied for 3D reconstruction for the median nerve of the hand-wrist and cubital fossa regions and the gastrocnemius nerve. This nerve internal fascicle 3D reconstruction technology would be helpful for aiding peripheral nerve repair and virtual surgery.

Peripheral nerves (PNs) are important pathways for the central nervous system to communicate to various parts of the body, receiving afferent sensory impulses and sending out efferent motor instructions. However, after their anatomical structures are damaged, their functions are affected. To better restore nerve functions, the nerve fascicles of the proximal stump at damage location should connect those of the distal stump with the same functional properties^1^. Based on the main functions, PN fascicles can be divided into those mainly with sensory fibers, those mainly with motor fibers, and those mixed fascicles with both sensory and motor fibers. However, the internal structures of nerve fascicles are complicated, and the relative position and number of nerve fascicles can change within a very short distance along a nerve, which is accompanied by differentiation and confluence of nerve fibers with different properties^2^.

As it is impossible to visually distinguish these PN fascicles by naked eyes, it remains a great challenge to reconnect the PN fascicles with the same functions during PN surgical repair^3^. To solve this problem, one possible way may be to establish a 3-dimensional (3D) nerve visualization system of PN fascicle types^4^. As 3D panorama images can be rotated and zoomed in any directions, they provide the possibility of reconstructing 3D graphics of the internal construction of a PN^5^. Work has been done previously on 2D cross-sections of PNs, which has, to some extents, revealed the structures and characteristics of nerve fascicles inside a PN. Based on their findings in the changing fascicle types at the distal end of a median nerve (one typical PN), Jabaley *et al*. (1980) drew one conceptual internal 3D topography diagram of the nerve^6^. To characterize median nerve internal anatomy, Watchmaker *et al*. (1991) continuously sectioned the nerve (around the distal forearm and hand region) along its 7-cm length and revealed anatomical features by nerve fascicle images of these serial hematoxylin and eosin (H&E)-stained sections. Furthermore, after image recognition and statistical analysis processing using computer programs developed, they studied the branching patterns of nerve fascicles by using the 3D reconstruction method^7^.

In the current work, we aimed to establish the key technologies involved to achieve a 3D reconstruction of PN fascicles as carried out on segments of peripheral nerves of a deceased adult person. The main objectives of this study were four folds: (1) to design templates for position line cross sections, to recognize the position line images and to register images of the nerve serial cross sections (or transform them into one coordinate system); (2) to extract images of the fascicle edges and merge them onto the nerve cross section images to obtain continuous and smooth edges of nerve fascicles; (3) to use multi-directional gradient and second-order gradient method combined with rough-means clustering method to achieve automatic functional type classification of nerve fascicles; and (4) to process the serial cross section images and achieve a 3D reconstruction of PN fascicles. Establishment of a 3D visualization system of nerve fascicle types will be of significance for peripheral nerve repair.

## Results

The entire procedure consists of six stages, namely, (1) serial section preparation and imaging, (2) position line recognition of section images, (3) image registration, (4) edge acquisition of nerve fascicles, (5) functional type recognition of nerve fascicles, and (6) 3D reconstruction of internal fascicles.

### Nerve serial section preparation and imaging

**Figure 1:**
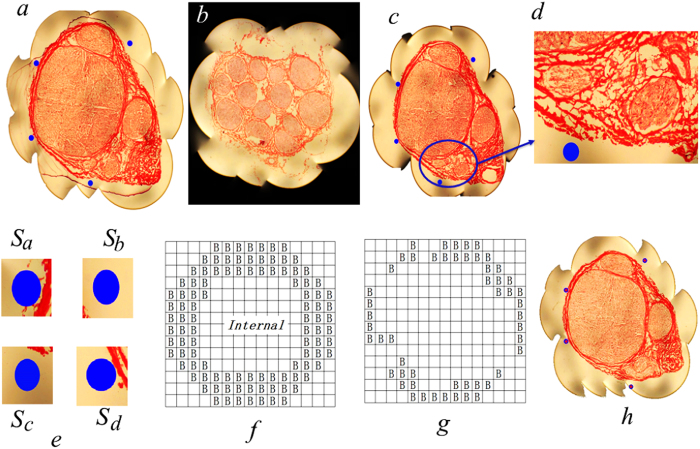
Position line recognition and edge acquisition of nerve fascicles in cross section images of median nerve. Images of randomly selected sections of (**a**) the cubital fossa median nerve, (**b**) the hand-wrist median nerve, (**c**) the first section, (**d**) the zoomed-in image of a region within (**c**). (**e**) Amplified cross-section images of the 4 position lines (the blue dots) within (**c**). (**f**) A template of a circular cross section image of 15 × 15 *pixels*, (**g**) a template of circle-like cross section image, and (**h**) the cross section image of the nerve with the central pixel coordinate after recognition of the position lines.

After a segment each of the median nerve in the cubital fossa region and in the hand-wrist region was dissected and embedded frozen together with four position lines (for easy identification), a total of 400 serial cross sections were obtained for each segment. After H&E staining, histological digital images were obtained, which were stitched using stitching software, with the stitched images having the resolution of 3488 × 2616 pixels. To improve the efficiency of computer processing, image resolution was reduced to 1024 × 768 pixels. Randomly picked cross-section images of the median nerve of cubital fossa and hand-wrist region are represented in Fig. 1a,b. Within these images, the dark blue circular dot images represent cross sections of position lines, and internal distinct areas formed together by discrete granular pixels represent nerve fascicles.

### Position line recognition of section images

To recognize the position line images and then to obtain their central pixel coordinates, the trained least square support vector machine (LS-SVM) method^8^ was used, with its parameters being optimized by using a genetic algorithm compiled. Cross-sections of the position lines were marked with letters *S*_a_, *S*_b_, *S*_c_ and *S*_d_ (Fig. 1c), and their magnified views were shown in Fig. 1d,e. It can be seen that position line cross-section contours were basically circular or circle-like. However, the distribution of cross-sectional pixels did not have a particular pattern within the interior of a cross-section. Furthermore, since the size of a position line is fixed, the diameters of the position line cross-sections within the slice image were found to fall within 12 × 12 to 15 × 15 pixels. After analyses of images of other slices, the same results were obtained.

With the position line cross-sectional images being within 12 × 12 to 15 × 15 *pixels*, a template of 15 × 15 *pixels* was constructed (Fig. 1f). One circle-like template was also designed by removing or modifying pixel positions labeled with “B” (Fig. 1g). With this template, a randomly selected area of 15 × 15 *pixels* from the nerve slice image can be determined as a circle or a circle-like object when the pixels of the edge of the intercepted area mostly fall within the area labeled with “B” (Fig. 1f). Then the central coordinate of the position line cross-section is assigned as the pixel coordinate of the eighth row and eighth column in the template. Thus, essentially, shape recognition of the cross-section of a position line is to determine whether edge shape of the intercepted area of 15 × 15 *pixels* image is circle-like or non-circular. After the position lines are recognized, the central pixel coordinate of the nerve section image can then be determined (marked “+”, Fig. 1h).

To verify recognition performance of the optimized LS-SVM method, this study has compared performances of this optimized method, the LS-SVM method with optional parameters and radial basis function (RBF) Neural Networks^9^ in recognizing 800 position line cross-sectional images in 200 nerve slices (Table 1). Performance of optimized LS-SVM method was found obviously better with improved accuracy than the latter two methods, with its misclassified error being 37 and 78% lower comparatively, respectively.

### Section image registration

**Figure 2:**
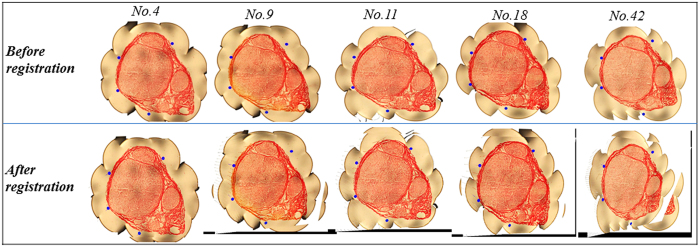
Registration results of 5 cross-section images randomly selected from the initial portion of the proximal median nerve of the cubital fossa. Left, before registration; right, after registration.

As a spatial normalization procedure for two section images, which could be acquired at different times or from different locations, the images need to be registered so to find an optimal transformation to align the two images in the same coordinate system. Previously, there have been several algorithms developed for this purpose, particularly in the area of medical image analyses^10^,^11^,^12^. In the current study, to improve the calculating efficiency, section image registration and alignment were implemented using the bilinear method^13^. Figure 2 displays results of registration of five section images randomly selected from the median nerve of the cubital fossa region. As it can be seen in the images after registration, the position lines were corrected to the same coordinate position of section images. The results showed that total registration time was 1380.2 s for the 400 acquired section images, with an average of 3.45 s per section.

### Edge acquisition of nerve fascicles

After registration of section images, edges of nerve fascicles need to be acquired before the fascicle functional types can be recognized. Since the fascicular groups converge and split while extending and thus their numbers in section images change, the fascicular group numbers in section images have to be acquired before obtaining the accurate edges of fascicular groups. For this purpose, a preprocessing step was implemented, and the unsupervised dynamic clustering method was employed. Furthermore, before edges of nerve fascicles can be acquired, the centers of nerve fascicles need to be obtained and pixels of nerve fascicles of each section image need to be processed by the clustering method. The clustering method must adapt to these constant changes (constant splitting and merging of fascicles) and to continuously adjust numbers and central positions of clusters.

#### Image preprocessing of nerve slice images

**Figure 3:**
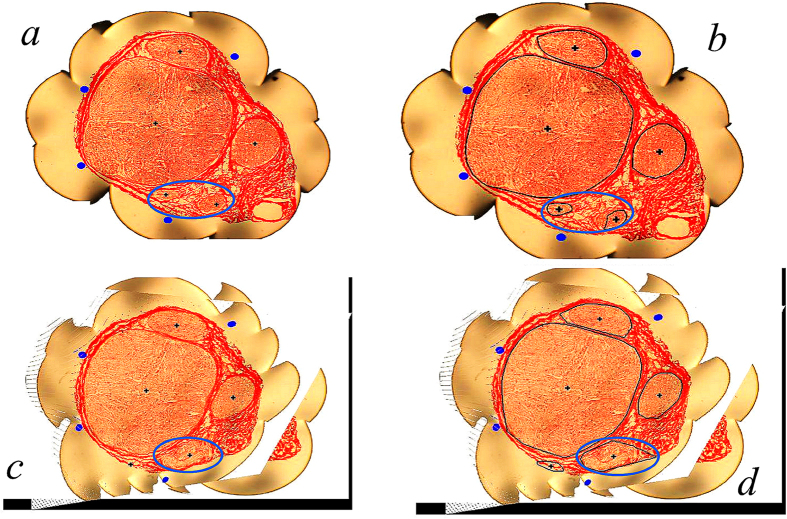
Image preprocessing and edge acquisition of median nerve slice images. (**a**) Image preprocessing result of No 1 nerve slice image; (**b**) image preprocessing result of No 42 nerve slice image; (**c**) extraction result for superimposing edges of nerve fascicle area in the first slice; (**d**) extraction result of No 42 slice image.

Image preprocessing outcomes of the first nerve slice section image are shown in Fig. 3a, in which the aggregation center of each nerve fascicle is marked with a “+”. Image preprocessing result of the 42^nd^ nerve section image is shown in Fig. 3b, in which the two nerve fascicles are shown merging into one. As seen from these examples, the numbers and central coordinates of the nerve fascicles can be accurately obtained by the dynamic clustering method with unknown number of clusters. In these two section images, other nerve fascicles have similar splitting and merging phenomena, and their corresponding numbers and central coordinates can be also accurately obtained by the adopted method.

Image preprocessing performance comparison has shown that the convergence speed of dynamic clustering method (16.7 s and 17.3 s respectively for 1^st^ and 42^nd^ sections) was obviously faster (70% on average) than the ant colony clustering method (28.5 s and 29.3 s for 1^st^ and 42^nd^ sections), which is one commonly used method for unknown numbers of clusters^14^. When the image preprocessing was carried out for the other section images, the same results were obtained.

#### Edge acquisition of nerve fascicles

After the center of each nerve fascicle was obtained and cluster processing for nerve fascicle pixels was completed, the edge of each nerve fascicle was then extracted. The first nerve section image (with the image pixel of 1024 × 768 *pixels*) was selected as an example for fascicle edge acquisition (Fig. 3c). Since pixel gray levels were found to vary considerably within the same fascicle (appearing not completely connected together in the location plane but being separated by many voids and discontinuities), the fascicle edges cannot be directly extracted by using the existing edge detection operator method or region growing method^15^,^16^,^17^. To be able to automatically extract fascicle edges, these voids and discontinuities have to be excluded. In addition, the extraction method adopted needs also to take the constant fascicle splitting/merging situation into account. In the current work, the nerve fascicle edges were extracted automatically by using the gradient vector flow (GVF)-Snake model^18^,^19^,^20^. Since the convergence speed of the classical GVF-Snake model is slow^21^,^22^,^23^, an improved GVF-Snake model was proposed in this study, in which the tangent energy part was removed from the GVF-Snake model since it was nearly zero and did not need to be calculated. With this improved GVF-Snake model, the snake (or active contour) was found to be able to converge rapidly to the edge of the target area. Similarly, fascicle edge extraction of the 42^nd^ nerve section image was carried out using the same method (Fig. 3d). By comparing Fig. 3c,d, it can be found that two nerve fascicles in the first slice (blue circle in Fig. 3c) are merging into one fascicle (blue circle in Fig. 3d). Thus, edges of nerve fascicles can be well extracted by using the proposed method in the study.

For testing the convergence performance of the improved GVF-Snake model, edge extraction results were compared for the 1^st^ and 42^nd^ section images as obtained by using the original GVF-Snake model vs by the improved GVF-Snake model (Table 2). It can be seen that the improved GVF-Snake model converged faster than the original GVF-Snake model, which on average, reduced the convergence time of edge extraction of each nerve fascicle by 2–3 s.

### Automatic recognition of nerve fascicle functional types

Since the internal fascicle types of the median nerve at the hand-wrist region are more complex than those in the cubital fossa, automatic recognition of functional types was carried out on section images of this region. To be able to achieve automatic functional recognition of nerve fascicles, feature description of their pixel neighborhood needs to be carried out, and a rational classification algorithm needs to be developed.

The pixel distribution of nerve fascicles in section images has some certain texture properties, namely, the pixels exhibit an irregularity in the local areas and a certain regularity on the whole. In the current study, to describe the features of the pixel neighborhood in nerve section images, a feature description method of multi-directional gradient and second-order gradient curves was proposed based on gray gradient analyses^24^,^25^ and as described in online Methods. In addition, also as described in online Methods, feature vectors (or sets of numeric features) of any pixels can be constructed, which together constitute the integrated image feature matrix.

After establishing feature matrix, every feature needs to be automatically classified. A good classification is the threshold classification automatically generated by calculation, rather than manually assigned. However, since the total pixel number of a nerve section image is up to one million, which would take a long time in the classification process if all pixels are used to construct the feature matrix, a rational algorithm needs to be designed for improving the operating speed and also having a relatively strong adaptability. In this study, the rough K-means algorithm^26^,^27^,^28^ was used to automatically recognize nerve fascicle functional types.

**Figure 4:**
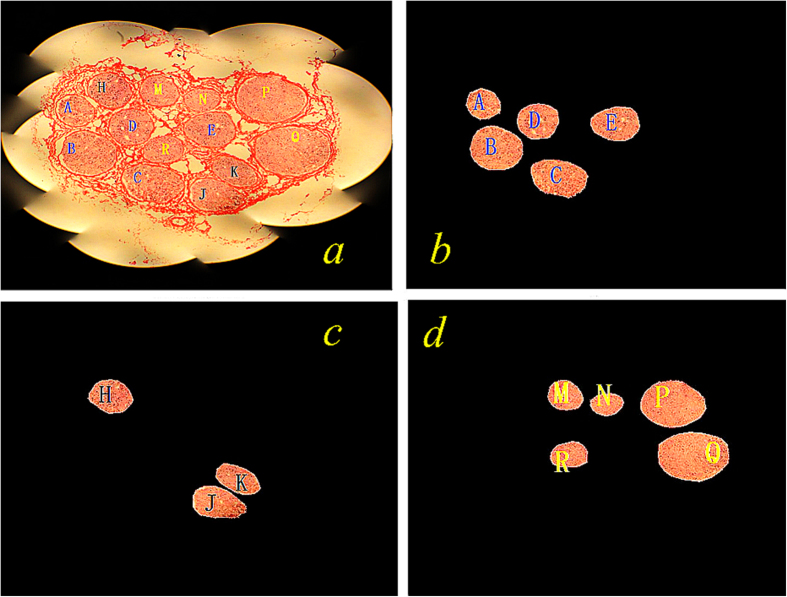
Functional recognition of median nerve fascicles. (**a**) One nerve slice image; (**b**) first recognition result; (**c**) second recognition result; (**d**) third recognition result.

For facilitating nerve fascicle type recognition, each nerve fascicle is marked with a letter (Fig. 4a). The tunica vaginalis of nerve fascicles in the sections have been removed by the staining method. In Fig. 4a, the fascicles mainly of sensory function appear relatively darker in color, those mainly of motor function relatively lighter, and those with mixed fascicles show lighter color pixel groups mixed with a small number of darker color pixel groups. After automatic functional type recognition with the scale of neighborhood being set at *r* = 11, the section images are shown in Fig. 4b–d. We can see that the pixels which represent the fascicular group areas are segmented, while most pixels which represent background areas are removed, and that Fig. 4b–d represent different types of fascicular groups with the dark pixel color. In Fig. 4b, fascicular groups marked “A”–“E” are of the same type. In Fig. 4c, groups marked “H”, “J” and “K” are of another type and other groups are separated to the same other type. The same results were obtained when the method was employed to other slice images.

Additionally, the current study has compared the accuracy and efficiency between our computer method and the manual method in recognizing the functional types of nerve fascicles of 200 slice images (Table 3). While the recognition accuracy of the manual way was a little better than computer’s method, the computer method was over 4 fold more efficient than the manual method. This study demonstrates that the fascicle feature extraction by multi-directional gradient and its second order gradient method is feasible, effective and efficient, and that our method for the automatic functional type recognition of nerve fascicles is simple, rapid and reliable.

### 3D reconstruction of median nerve fascicles

**Figure 5:**
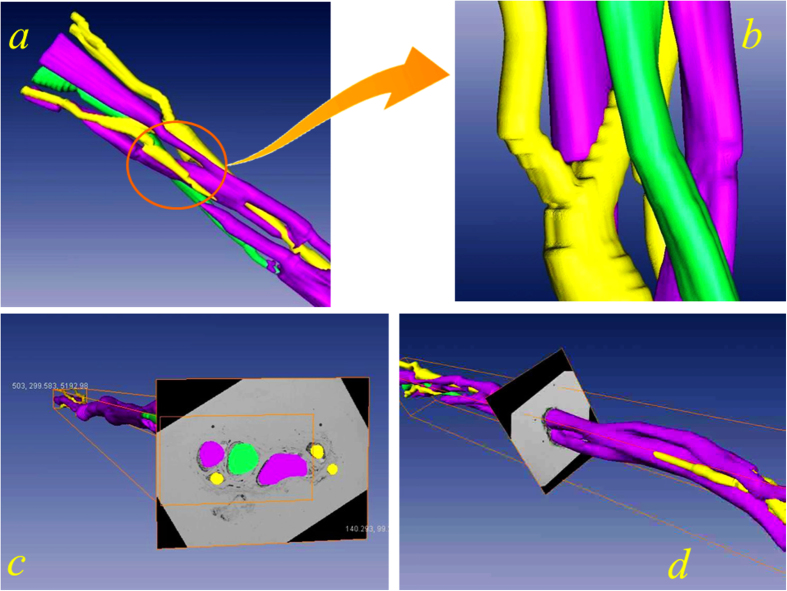
3D reconstruction of median nerve fascicles. (**a**) A 3D model of median nerve at an arbitrary position, showing some changing patterns of different functional fascicles. Purple – mixed nerve fascicles, green – motor nerve fascicles, yellow – sensory nerve fascicles. (**b**) One transverse cross section image, which shows that the position outline is consistent with the original 2D image.

After the above described procedures for position line recognition, image registration, fascicle edge acquisition and functional type recognition, the resulting section images can then be used to perform the 3D reconstruction. For 3D reconstruction, purple, green and yellow drawings were used to represent fascicles of different functions as well as to distinguish the cross and fusion between the nerve bundles. The reconstructed results of the median nerve are presented in Fig. 5, which shows a 3D model of the nerve in an arbitrary position (Fig. 5a) and the different natures and combinations of nerve fascicles (Fig. 5b). By adjusting the related parameters of software, we can control the orientation of the image presentation, angles and so on, and we can implement the human-computer interaction arbitrary 3D rotation, which shows a clear and strong sense of the entity as a whole. From this 3D model, it can be seen that nerve fascicles can cross and merge along its length, demonstrating and confirming the intuitive complex variations of the different functional fascicle types of the median nerve. In addition, it confirms that a peripheral nerve includes those nerve fascicles mainly of sensory function, those of motor function, and those mixed nerve fascicles with sensory and motor functions.

### Experiments on other nerve specimens and analyses of experimental results

**Figure 6:**
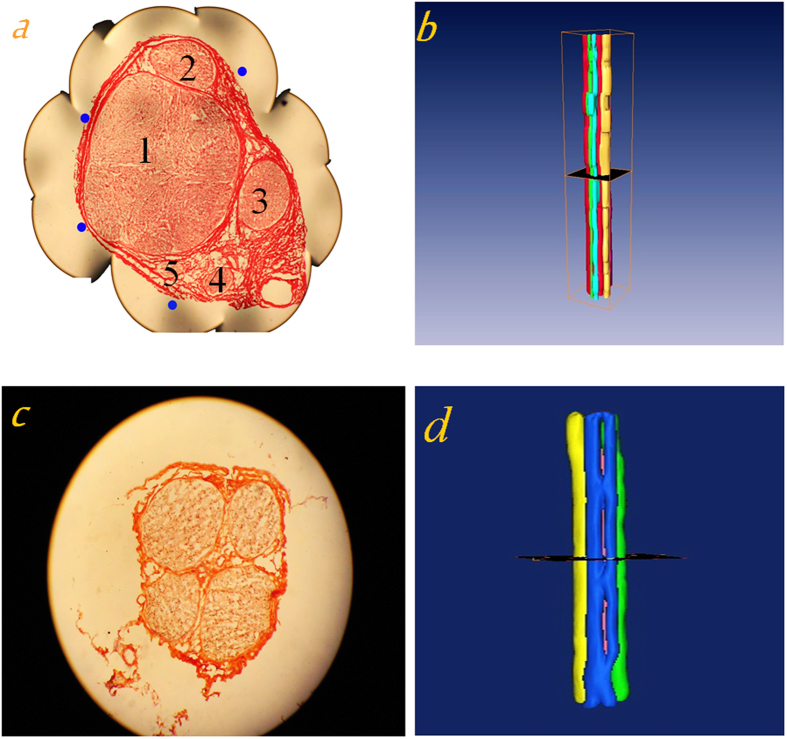
3D reconstruction results of median nerve of cubital fossa region and gastrocnemius nerve. (**a**) A section image selected randomly and (**b**) 3D reconstruction result for median nerve of cubital fossa region; (**c**) a section image selected randomly and (**d**) 3D reconstruction result for the gastrocnemius nerve.

In addition, the entire procedure described above was also applied to the hand-wrist region of median nerve and the gastrocnemius nerve with results shown in Fig. 6. Figure 6a,c show a section image each selected randomly from median nerve of cubital fossa region and the gastrocnemius nerve; and Fig. 6c,d illustrate the 3D reconstruction results of the median nerve of cubital fossa region and the gastrocnemius nerve.

Furthermore, the 3D reconstruction parameters were compared for these different nerve specimens (Table 4). During the preparation for nerve serial section images, the success rates of staining the median nerve sections of cubital fossa region and hand-wrist region were 86.4% and 87.3%, respectively, and that of the gastrocnemius nerve was 87.5%. During the segmentation of nerve fascicles, the mean error rates of the median nerve of cubital fossa region and hand-wrist region were 5.7% and 8.8% respectively, and that of gastrocnemius nerve was 11.3%. During the recognition of fascicle functional types, the mean error rates were 8.1%, 8.7%, and 1.2, respectively for the three specimens (being lower for the gastrocnemius nerve as it contains mainly motor fibers). The similarity between the 3D reconstruction result and the actual fascicle situation of the specimen was 94.3%, 91.2%, and 88.7%, respectively for the three different specimens. These results suggest that the 3D reconstruction accuracy is quite similar among these different specimens.

## Discussion

It has remained a great challenge to butt the nerve fascicles of the same function in peripheral nerve repair. To solve this problem, one possible way is to establish a 3D visualization system of nerve fascicles. In recent years, the computer 3D reconstruction technique using the continuous serial sections is becoming one of the main methods to study soft tissue internal structure. In this technique, 2D images of a biological tissue structure are firstly obtained through continuous sectioning and then entered into a computer and processed using a specially designed 3D software, which produces a 3D solid shape structure^29^. In fact, the 3D reconstruction technology of 2D rigid cross-sections is relatively mature, including techniques for magnetic resonance imaging (MRI), diffusion tensor imaging (DTI), distinguished diagnostic Imaging (DDI) and diffusion weighted imaging (DWI). However, although these 2D rigid cross section-based 3D reconstruction techniques can provide technical references, it is difficult to achieve 3D visualization of nerve internal fascicle types by using these techniques due to the small cross-sectional areas, complex internal structures, and a great variation in functional types of nerve fascicles along the length of the nerve^30^,^31^,^32^,^33^. Therefore, the 3D visualization of nerve internal fascicles has to be obtained by the non-rigid 2D images of tissue sections. Zhang *et al*. (2009) had investigated 3D reconstruction of fascicular groups inside a segment of common peroneal nerve based on internal micro-dissection and histological anatomy^34^. Although some related studies have also existed for 3D nerve reconstruction^4^,^34^,^35^, and preliminary studies were reported in Chinese literatures on nerve fascicular edge extraction^36^ and functional type recognition^37^, the key technologies for different stages involved for 3D reconstruction of peripheral nerve internal fascicle types have not been reported previously.

Here, we have established key techniques to achieve 3D reconstruction of nerve internal fascicles types. Firstly, after continuous, serial histological section images are obtained, the numbers of nerve fascicles and the central positions of nerve fascicles on section images are determined, and pixel groups of nerve fascicles are extracted. Secondly, the nerve fascicle edges are extracted one by one by using the envelope method^18^,^19^,^20^, and the obtained edges are merged onto the original section images so to obtain continuous and smooth edges for the nerve fascicles. Thirdly, this study has achieved automatic and consistent classification of nerve fascicles in different section images by using image processing and pattern recognition methods. Finally, the current study has successfully achieved 3D reconstruction of peripheral nerve internal fascicles types.

To carry out slice image registration, cross-sections of position lines of every slice image need to be recognized for obtaining their central pixel coordinates which can be used as control points to complete slice image registration. Here, the LS-SVM method was adopted to recognize cross-sections of position lines. In addition, parameters of LS-SVM were optimized by using a genetic algorithm following training of the LS-SVM method by a designed training set of templates, testing and obtaining misclassified errors of LS-SVM by a test set of templates. By using our optimized LS-SVM method, cross-sections of position lines can be recognized as a whole; and by using this recognition method, the efficiency and accuracy of recognition are improved. Comparison of recognition methods have shown that the recognition performance of our optimized LS-SVM method was significantly better than the original LS-SVM method and the RBF Neural Networks method. This is important as a high recognition accuracy lays the foundation for subsequent image registration.

The nerve fascicles constantly split and merge in the extension process, and thus section images need to be preprocessed prior to image registration. To automatically get the correct number and central positions of fascicle clusters of section images, unsupervised clustering method was adopted^14^,^38^,^39^, which was shown to have a faster convergence speed than the more commonly used ant colony clustering method for unknown numbers of clusters.

In nerve section images, the fascicles appeared as large areas with dark pixel clusters that gathered together and with discrete granular distribution. Due to inconsistency in the pixel color depth even within the same fascicle, the edge of a nerve fascicle cannot be automatically extracted by using the direct color space method^40^,^41^,^42^. In addition, since pixel gray levels vary considerably within the same fascicle (being separated by many voids and discontinuities), edges of the nerve fascicles cannot be obtained directly by adopting some existing methods, e.g. edge detection operator method and region growth method^15^,^16^,^17^. Here, after testing with the classical GVF-Snake model with normal and tangent energy part, it was found that the model can be simplified because the tangent energy part is nearly zero while the edge is enveloping the fascicle. Thus in the current study, the improved GVF-Snake model (in which the tangent energy part of GVF-Snake was removed) was employed to envelop the edge of each nerve fascicle, and the experiments showed that the improved GVF-Snake model was effective to extract edges of nerve fascicles.

Since the internal structures of nerve fascicles are complicated, and the relative position and number of nerve fascicles can change within a very short distance along a nerve (accompanied by differentiation and confluence of nerve fibers with different properties), it is difficult to visually distinguish the three functional types of nerve fascicles in the slice images. Additionally, the feature extracting methods mentioned in literatures cannot distinguish the functional types of nerve fascicles in the slice images. In the current study, we have used a multi-directional gradient and its second order gradient to extract the features of nerve fascicles in slice images and then to employ the rough k-means algorithm to classify the fascicle types automatically since the parameters in rough k-means algorithm can be calculated through algorithm instead of manual setting. Our experimental results revealed that (1) the features extracted through multi-directional gradient and its second order gradient can clearly distinguish fascicle functional types in slice images; and (2) the parameters of the feature extracting method hardly influence the accuracy rate in recognizing the fascicle functional types, indicating a good adaptability of this method.

In this study, this technique has been successfully applied to carry out 3D reconstruction for the median nerve of the hand-wrist region, the median nerve of cubital fossa region, and the gastrocnemius nerve. In all three cases, the reconstruction results are quite similar to the actual fascicle situation of the nerve specimens. This demonstrates the reasonable accuracy, validity, and efficacy of the method developed, suggesting that the method can potentially be adapted to reconstruct nerve fascicles of various peripheral nerves of a human body.

The method described in this study is quite different from the traditional way of 3D nerve reconstruction (Table 5), which uses Mimics or Amira software for reconstruction from the images acquired from computed tomography, magnetic resonance imaging or diffusion tensor imaging equipment. The traditional way can only reconstruct large nerves as it is limited by the resolution of images acquired. The traditional way is, however, real time, rendering its efficiency obviously better than that of our method and its clinical application for conditions involving large nerves. While the traditional way cannot automatically recognize nerve fascicle types and cannot exhibit the complicated internal structures of nerve fascicles, their crossing and merging along the nerve’s length, our method can do all these, which demonstrates a better specificity for our method. This feature would be particularly important for surgical navigation and guiding functional nerve bundle reconnection during surgical nerve repair. It is also worth mentioning that, due to its ability to reveal internal nerve structure, our method has now been adopted for clinical education at The First Affiliated Hospital of Sun Yat-sen University and Shenzhen Sixth People’s Hospital in China.

While the current technique was developed using normal nerve specimens (non-injured, non-distorted and cylinder-like), future studies are required to investigate whether this technique can be adopted to reveal internal detailed structures of defected/injured nerves or repairing nerves. Further adaptation of this technique and its potential successful application in abnormal nerves may render its likely value in meeting the current challenges for performing non-invasive diagnosis of a nerve injury or other nerve pathological conditions, for guiding minimally invasive nerve surgery and for surgical navigation for reconnection of different nerve bundles. While 3D reconstruction results are expected to be different between normal and damaged nerves, this technique described and the data generated in the current study will provide an important basis and reference data for these future studies. In addition, this 3D model method will be potentially useful to help us to understand the nerve complex internal information and functionality and to set up virtual 3D nerve internal fascicle databases for different peripheral nerves and at different regions, such databases will ultimately be useful for developing a non-invasive 3D nerve fascicle reconstruction approach for diagnostics of nerve injury and for guiding nerve repair surgery.

In summary, in this study, the key technologies of 3D reconstruction of peripheral nerve internal fascicular groups were established through actual cases of peripheral nerves, including serial section preparation and imaging, image position line recognition and image registration, nerve fascicular edge acquisition, fascicle functional type recognition and 3D reconstruction of nerve fascicles of different types. After the LS-SVM method was trained and optimized by a designed training set, its accuracy in recognizing cross sections of position lines is about 91.75%. To align and register nerve section images, the bilinear method was found efficient and its accuracy rate satisfies the requirement of 3D reconstruction. For extracting the edges of nerve fascicles, the GVF-Snake method was found effective and efficient, enabling the contours of fascicular groups in nerve slice images to be smooth and continuous, and most slice images can be processed within 120 seconds. The current study has also proposed an approach to recognize the fascicular groups by multi-direction gradient and corresponding 2^nd^ derivative gradient of gray level, and this approach was found to recognize types of fascicular groups accurately and efficiently, with the recognizing results not related with the parameters. Finally, 3D reconstruction results of nerve internal fascicular groups are close to the real nerve internal structure. Therefore, data of this study suggest that the key technology described here can potentially be used to achieve accurate 3D reconstruction of the peripheral nerve internal fascicular groups, which can reveal the complicated anatomical nerve fascicle structure and changes in fascicles along its length. To be able to achieve 3D reconstruction of nerve internal fascicles of different functional types of limbs will have a great clinical value. Apart from its potential in aiding accurate internal positioning of nerve fascicles for nerve repair, the establishment of a relatively complete and accurate virtual 3D nerve internal fascicle database will be a valuable education tool, provide important match options and guidance for nerve tissue engineering, artificial nerve creation, rational choices/designs for various nerve translocation and transplantation, for diagnosis of peripheral nerve disorders, for nerve surgical repair and for virtual surgery.

## Methods

### Median nerve sampling, serial sectioning, and imaging

The median nerve in the cubital fossa region was dissected from an unfrozen adult cadaver within 24 hours of death with informed consents obtained from all subjects (the subject prior to death and deceased person’s family), and the dissection and the use of the median nerve were carried out in accordance with the approved guidelines and were approved by the Human Research Ethics Committee of Sun Yat-sen University (Guangzhou, China). After removing other tissues (e.g. fat) around the nerve, the nerve specimen was placed on a small piece of soft wooden board, and both ends were fixed on the board by pins to prevent the nerve from curling and to maintain it straight. Four adult female hairs (used as position lines) were used to fix around the nerve specimen, and specimen and hairs were embedded in OCT (optimal cutting temperature) cryostat embedding medium. Then the nerve specimen was stored in −80 °C and taken out for cryostat sectioning one week later. The median nerve specimen was firstly cut into 20 mm-long segments, which were then sliced in a cryostat microtome to obtain serial cross sections of 15 μm in thickness and 0.5 mm apart. The same approach was used to prepare serial sections of the median nerve of the hand-wrist region. After being stained by H&E, all sections were viewed by Z61 stereo microscope with a MSHOT MD90 microscope digital imaging device, and digital images (in JPG format) were acquired (under 1.5 × 4 magnification).

### Position line recognition of slice images and image registration

To recognize the position line images and then to obtain their central pixel coordinates, the trained LS-SVM method was used, with parameters of LS-SVM being optimized by using a genetic algorithm, with the computer programming being compiled using MATLAB 2009B (The MathWorks, Inc., Natick, MA) in a computer with i2-2.2G CPU and 2G memory.

### Edge acquisition of nerve fascicles

Firstly, the number of nerve fascicles and the central positions of nerve fascicles were obtained, and pixel groups of nerve fascicles were extracted. Secondly, the nerve fascicle edges were extracted one by one by using an improved GVF-Snake model, in which the tangent energy part was removed from the GVF-Snake model. Finally, the obtained edges were merged onto the original section image so to obtain continuous and smooth edges for the nerve fascicles.

### Automatic recognition for functional types of nerve fascicles

To describe the features of the pixel neighborhood in nerve slice images, the multi-directional gradient and second-order gradient method was used. Image f has M × N pixels with the RGB color model, and the pixel of image coordinate (m,n) is randomly selected. The neighborhood of 2*r* + 1 is *S*, *r* ≥ 5, and the multi-direction gradient of this pixel in the neighborhood at 0° direction is:


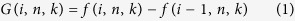


where, *i* = (*m* − *r* + 1) … (*m* + *r*), *k* = 1, 2, 3 represents the color layer *R*, *G* and *B*, respectively.

The second-order gradient of this pixel in the neighborhood at 0° direction is:


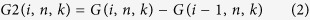


where, i = (*m* − *r* + 2) … (*m* + *r*).

According to Eq. (1) and (2) above, feature vectors (or sets of numeric features) of any pixels within a neighborhood in the image can be constructed, and the feature vectors together constitute the integrated image feature matrix. To obtain the fundamental and harmonic cycles of multi-directional gradient and second-order gradient curves, Fourier transformation method would require a large number of computations. Thus, to describe curves for a second order gradient and a certain direction-gradient, the current study used the maximum amplitude and second large amplitude of multi-directional gradient and second-order gradient curves, and the average peak time interval. Thus, for a randomly selected pixel neighborhood, there are four directions for the gradient and four directions for the second order gradient. Each gradient can be described by three parameters, and there are three color components when using the RGB color model. Therefore, the number of feature vectors for describing the randomly selected pixel neighborhood is 8 × 3 × 3 = 72. A 72-dimensional feature matrix can be obtained when all feature vectors of randomly selected pixels are integrated together.

To examine the accuracy and efficiency in recognizing the functional types of nerve fascicles of 200 slice images, first, functional types of nerve fascicles in each slice image were manually marked one by one, and the elapse time was measured. Then we checked manually whether the functional marking of each nerve fascicle was correct or not. Next, the functional types of nerve fascicles of 200 slice images were recognized automatically by the computer method described above with the elapse time being also recorded. The corresponding parameters were set to *r* = 11 and the number of pixels selected randomly was 20000.

### 3D Reconstruction of median nerve fascicles

3D reconstruction of median nerve fascicles was implemented using software Amira 4.1 (TGS Co. Ltd, Bordeaux, France). Fascicle functional types were entered into different data channels. Each channel was processed first individually and then all channels were combined to achieve the whole 3D reconstruction displaying. With the computer configured to simplify contour line appropriately, the shaded surface display (SsD) method was used to display the reconstructed 3D model.

### Validating experiments with other nerve specimens

For validating the technique developed, the median nerve of cubital fossa region and the gastrocnemius nerve were also chosen for 3D reconstruction. In a randomly selected image of median nerve of cubital fossa region, each fascicle was marked with a number. For a clearer display of fascicles during reconstruction, fascicles marked with “1”, “2”, “3”, “4”, or “5” were set to red, purple, yellow, blue, or green color, respectively (Fig. 6a).

## Additional Information

**How to cite this article**: Zhong, Y. *et al*. Three-dimensional Reconstruction of Peripheral Nerve Internal Fascicular Groups. *Sci. Rep*. **5**, 17168; doi: 10.1038/srep17168 (2015).

## Figures and Tables

**Table 1:** Comparison of recognition performance by different recognition methods.

Recognition method	Kernel function parameter *σ*^2^	Adjustable parameter *γ*	Misclassified error (%)
LS-SVM with optimized parameters	4.71	224	8.25%
LS-SVM with optional parameters	2	100	11.375%
RBF Neural Networks	—	—	14.75%

**Table 2:** Convergence rate comparison of edge extraction for the 1^st^ and 42^nd^ section images with the original GVF-Snake model and improved GVF-Snake model.

Processing method	No. 1 section	No. 42 section
Original GVF-Snake	91. 57 s	87.23 s
Improved GVF-Snake	67.38 s	67.51 s

**Table 3:** Processing time and accuracy comparisons between manual and computer processing.

Processing mode	Accuracy rate	Time consumed
Manual	99.7%	42 hrs
Computer	91.6%	9.5 hrs

**Table 4:** Parameter analysis of 3D reconstruction results in different specimens.

	Median nerve of cubital fossa region	Median nerve of hand-wrist region	Gastrocnemius nerve
Image preparation	463 sections were obtained; 400 sections having complete morphology and clear texture were chosen (86.4%)	458 sections were obtained; 400 sections having complete morphology and clear texture were chosen (87.3%)	435 sections were obtained; 380 sections having complete morphology and clear texture were chosen (87.5%)
Mean error during segmentation of fascicles	5.7%	8.8%	11.3%
Mean error during recognition of the fascicle functional types	8.1%	8.7%	1.2%
Similarity between 3D reconstruction results and actual fascicles of the specimens	94.3%	91.2%	88.7%

**Table 5:** Comparing our method with traditional 3D reconstruction way.

	Clarity of fascicles in section image	Registration method	Method to acquire the edges of fascicles	Recognition of fascicle functional types	Similarity between reconstructed results and actual fascicles	Operation time required	Repeatability
Our method	Clear	Our algorithm	Our algorithm	Our algorithm	80–95%	Complex operation, needing a long time	80–90%
Traditional 3D reconstruction method	Unclear	No registration	Manual	Without this function	Cannot judge the similarity degree	Easy to operate	99%
